# Associations between intronic non-B DNA structures and exon skipping

**DOI:** 10.1093/nar/gkt939

**Published:** 2013-10-22

**Authors:** Zing Tsung-Yeh Tsai, Wen-Yi Chu, Jen-Hao Cheng, Huai-Kuang Tsai

**Affiliations:** ^1^Institute of Information Science, Academia Sinica, Taipei, 115, Taiwan, ^2^Bioinformatics Program, Taiwan International Graduate Program, Academia Sinica, Taipei, 115, Taiwan and ^3^Institute of Biomedical Informatics, National Yang-Ming University, Taipei, 112, Taiwan

## Abstract

Non-B DNA structures are abundant in the genome and are often associated with critical biological processes, including gene regulation, chromosome rearrangement and genome stabilization. In particular, G-quadruplex (G4) may affect alternative splicing based on its ability to impede the activity of RNA polymerase II. However, the specific role of non-B DNA structures in splicing regulation still awaits investigation. Here, we provide a genome-wide and cross-species investigation of the associations between five non-B DNA structures and exon skipping. Our results indicate a statistically significant correlation of each examined non-B DNA structures with exon skipping in both human and mouse. We further show that the contributions of non-B DNA structures to exon skipping are influenced by the occurring region. These correlations and contributions are also significantly different in human and mouse. Finally, we detailed the effects of G4 by showing that occurring on the template strand and the length of G-run, which is highly related to the stability of a G4 structure, are significantly correlated with exon skipping activity. We thus show that, in addition to the well-known effects of RNA and protein structure, the relative positional arrangement of intronic non-B DNA structures may also impact exon skipping.

## INTRODUCTION

Alternative splicing, the selective removal of introns and reconnection of exons by multiple processes, is generally accepted to be coupled with transcription. Two prevailing models have been proposed to explain the coupling between alternative splicing and transcription: the recruitment model and the kinetics model (1). The recruitment model suggests that splicing factors are recruited to transcription sites by the transcription machinery, including RNA polymerase (2), the Mediator complex (3) and coactivators (4), thereby executing their functions. The kinetic model states that the rate of RNA polymerase II (Pol II)-mediated elongation influences splicing outcome (1). For example, sequences flanking alternatively spliced exons could be bounded by small interfering RNA, and the binding event leads to changes in chromatin structure, reduction of Pol II elongation efficiency and eventually the production of different splice variants (5). Numerous other studies have found that changing Pol II elongation rate resulted in different splice products (6–10), further supporting kinetic coupling. Although the kinetic coupling model has thus provided a convincing explanation for the production of splice variants, our understanding of the specific mechanism that describes how Pol II elongation influences alternative splicing is still far from being comprehensive.

Most researches on splicing regulation are from the perspectives of RNAs and proteins. There is, however, increasing evidence that noncanonical DNA structures, coined non-B DNA structures, have functional roles in several biological processes, such as gene expression regulation (11,12) and chromosomal rearrangements (13). Particularly, G-quadruplex (G4), a four-stranded non-B DNA structure characterized by multiple G-runs (i.e. consecutive guanines longer than three nucleotides) and short loops (14), has recently been observed to impede Pol II activity during transcription (15). Moreover, frequent presence of G4 immediately after the 3′ end of genes suggests that G4 may be involved in the termination of Pol II transcription (16). Coincidentally, Pol II has been reported to pause at 3′ splice sites and 3′ most exons to ensure splicing before releasing the transcript (17,18). These observations therefore lead to the speculation that G4 is associated with alternative splicing.

Kostadinov *et al.* observed that G4 frequently exists near alternative splice sites (19), which reside in the flanking regions of exons. Furthermore, in human p53, presence of G4 in the third intron could regulate the splicing of the second intron (20). These studies indicate the possibility that G4 plays a role in producing alternatively spliced transcripts. However, the detailed involvement of G4 is elusive, and whether the association between G4 and alternative splicing is universal in mammals remains unclear. In addition to G4, other non-B DNA structures (i.e. cruciform DNA, triplex DNA, slipped DNA and Z-DNA) have also been reported to be abundant in the genome (21–25). These non-B DNA structures have the capability to arrest the transcription process (26–28), possibly by influencing Pol II (29). As recent literature suspects that G4 may reduce Poll II activity through structural blockage (15,19,20), it is worth investigating whether these other non-B DNA structures (with the potential to form a structural obstacle for Pol II) are also associated with alternative splicing, and if so, whether these non-B DNA structures have similar properties as G4 in affecting alternative splicing.

In this study, we conducted a genome-wide investigation to study the associations of these above-mentioned non-B DNA structures with exon skipping, the most common mode of alternative splicing. To the best of our knowledge, this is the first genome-wide investigation of the associations between non-B DNA structures and exon skipping across mammals. By analyzing the distributions of non-B DNA-forming sequence motifs in human and mouse genomes, we demonstrated that the existence of non-B DNA structures at flanking sequences of alternatively spliced exons is significantly correlated to exon skipping. Moreover, our results showed that the associations of non-B DNA structures with exon skipping might be influenced by the position of non-B DNA structures relative to the exon. Interestingly, we found that the associations between non-B DNA structures and exon skipping may be species-specific. In human, non-B DNA structures tend to have a stronger association with exon skipping in the upstream intron than the downstream intron. In mouse, conversely, non-B DNA structures in the downstream intron have stronger association than in upstream. In addition, we found that the length of G-run, a factor previously thought to enhance the stability of G4 structure (30), is correlated with the exon skipping activity. Altogether, we show that, on top of the well-studied RNA and protein structure, the position of these non-B DNA structures also might be critical in splicing determination.

## MATERIALS AND METHODS

### Data collection and non-B DNA structure identification

NCBI36 human assembly and NCBI m35 mouse assembly were used in this study. We collected introns of cassette exons (skipped exons), the most common form of alternative splicing, from the Alternative Splicing and Transcript Diversity database (ASTD database, release 1.1, build 9) (31). To avoid potential bias and to simplify the analysis, duplicated entries, overlapped entries or entries with more than three exons were removed. Finally, 18 533 cassette exon cases in human and 9477 cases in mouse were used in this study. We also collected 106 499 human and 127 372 mouse constitutive exons (i.e. all the annotated exons except alternatively spliced exons such as cassette exon, mutually exclusion, exon isoform, intron isoform and intron retention) from the ASTD database for comparison.

Following Cer *et al.* (32), occurrences of five non-B DNA structures (cruciform DNA, triplex DNA, slipped DNA, G4 and Z-DNA) were identified through scanning these introns using the sequence motifs and criteria as described in Table 1. Notably, overlapped occurrences were excluded from our analyses to avoid statistical bias. Moreover, to avoid potential bias on the association with exon skipping from polypyrimidine tract to triplex DNA structure, which is also pyrimidine-rich, we excluded the identified triplex DNA that overlapped with polypyrimidine tract in the subsequent analyses. The polypyrimidine tract was defined as a 20-bp region containing no more than five purines and located within a 40-bp region of the 3′ end of an intron (33).

**Table 1.** Sequence motifs and search criteria of five non-B DNA structures

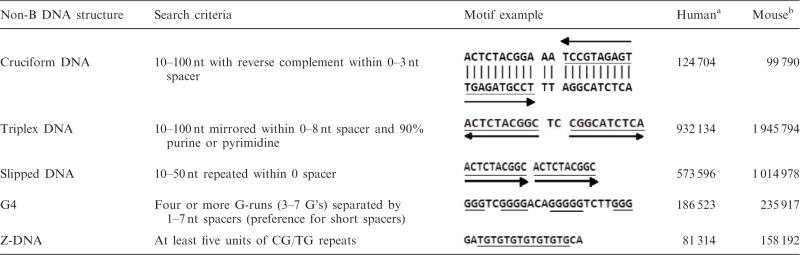

^a^Number of occurrences in analyzed introns of human genome.

^b^Number of occurrences in analyzed introns of mouse genome.

### Statistical analysis

For each non-B DNA structure, all the cassette exons with non-B DNA occurrences were grouped based on the region (in upstream or downstream intron) of non-B DNA structures. We calculated the proportion of alternative skipped exons in each group, and compared the proportions with the background exon skipping probability. The background exon skipping probability was defined as the proportion of alternative skipped exons in all the exons that have both upstream and downstream introns. The probability is 0.148 and 0.069 for human and mouse, respectively. To investigate whether occurring on the different strand plays into the association between non-B DNA structure and exon skipping, the occurrences of G4 in template or non-template strands were discussed independently.

Similarly, we studied the association between exon skipping and G-runs within G4 motifs. The length of the longest G-run within G4 motifs in every intron was calculated. The G4-containing cassette exons were then grouped by G-run occurrence strand (on the template strand or non-template strand), region (on the downstream or upstream intron) and the G-run length. Similarly, the proportions of alternative skipped exons were calculated and compared with the background exon skipping probability.

The potential associations between non-B DNA occurrences and exon skipping are evaluated by partial Pearson correlation coefficient, controlling for intron length. Partial correlation measures the relationship between two variables, *x* and *y* (e.g. non-B DNA occurrence and exon skipping), when conditioning on other variables, *z* (e.g. intron length). The partial correlation of *x* and *y* adjusted for *z* is defined as the correlation between residuals of *x* and *y* after regressed on *z*.

To evaluate the significance of the association between exon skipping and non-B DNA structure, we also compared the occurrence frequencies of non-B DNA on introns of alternative and constitutive exons. The numbers of introns having non-B DNA occurrences for alternative and constitutive exons were calculated and compared by one-sided two-sample proportion test.

Furthermore, to assess the relative contributions of the five non-B DNA structures to exon skipping, we constructed the following multiple logistic regression model,

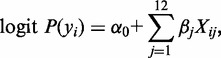

where *y_i_* = 1 represents the *i*-th sample that is alternatively spliced and *y_i_* = 0 represents the *i*-th sample that is not; *X_ij_* denotes the value of *j*-th feature of *i*-th sample; *α_0_* is an intercept term and *β_j_* is the effect of the *j*-th variable. The 12 variables are (i) upstream intron length, (ii) downstream intron length, (iii) five variables for the number of non-B DNA occurrences in the upstream intron and (iv) five variables for the number in the downstream intron. We evaluated the model and the individual contribution of the predictors based on the regression coefficients, which represent the impact of the logit for each unit change in the predictor. Wald z-statistic was used to assess the significance of each predictor. All the statistical analyses were conducted in the R statistical package version 2.15.1.

## RESULTS

### Non-B DNA occurrences are significantly associated with exon skipping

We identified the motif occurrences of the five non-B DNA structures (G4, cruciform DNA, triplex DNA, slipped DNA and Z-DNA; see Table 1) in the flanking introns of cassette exons to study their relationship with exon skipping (detailed in ‘Materials and Methods’ section). Our investigation considered the region (upstream or downstream) of all non-B DNA structures, and the strand (template or non-template) of G4. Both region and strand were not explored in previous studies (15,16). For each combination of non-B DNA occurrence, strand and region, we evaluated the partial Pearson correlation between non-B DNA occurrence and exon skipping adjusting for intron length (detailed in ‘Materials and Methods’ section), which has been indicated to be associated with exon skipping (34,35). The results show that, in human, most non-B DNA structures are more significantly correlated with exon skipping in upstream introns than in downstream introns (Table 2). However, non-B DNA structures in mouse, particularly G4, show more significant correlations to exon skipping in downstream introns rather than upstream. Interestingly, in both human and mouse, the correlation with exon skipping of G4 is significant only when residing on the template strand. Likewise, cruciform DNA and slipped DNA in upstream introns show significant correlations with exon skipping, but their significance drop when occurred in downstream introns. In human, only triplex DNA remains unaffected by occurrence region, showing consistently significant associations to exon skipping in every position. These observations suggest that the associations of non-B DNA structures with exon skipping vary in different positions and in different species.

**Table 2.** Association of non-B DNA structures with exon skipping when adjusting for intron length

Region	Non-B DNA structure	*P*-value^a^ (human)	*P*-value^a^ (mouse)
**Upstream**	Template G4	3.09 × 10^−19^ (***)	1.1 × 10^−4^ (**)
	Nontemplate G4	0.027	0.192
	Cruciform DNA	8.46 × 10^−5^ (***)	0.005 (*)
	Slipped DNA	4.9 × 10^−5^ (***)	0.002 (*)
	Triplex DNA	6.69 × 10^−6^ (***)	0.302
	Z-DNA	3.82 × 10^−3^ (*)	0.941
**Downstream**	Template G4	0.004 (*)	2.41 × 10^−9^ (***)
	Nontemplate G4	0.636	1.97 × 10^−4^ (**)
	Cruciform DNA	0.001 (*)	0.007 (*)
	Slipped DNA	0.002 (*)	0.026
	Triplex DNA	2.54 × 10^−10^ (***)	0.002 (*)
	Z-DNA	0.007 (*)	0.998

^a^Partial Pearson correlation coefficient test was used to obtain the *P*-value. *P < 0.01, **P < 0.001 and ***P < 0.0001.

**Figure 1.**
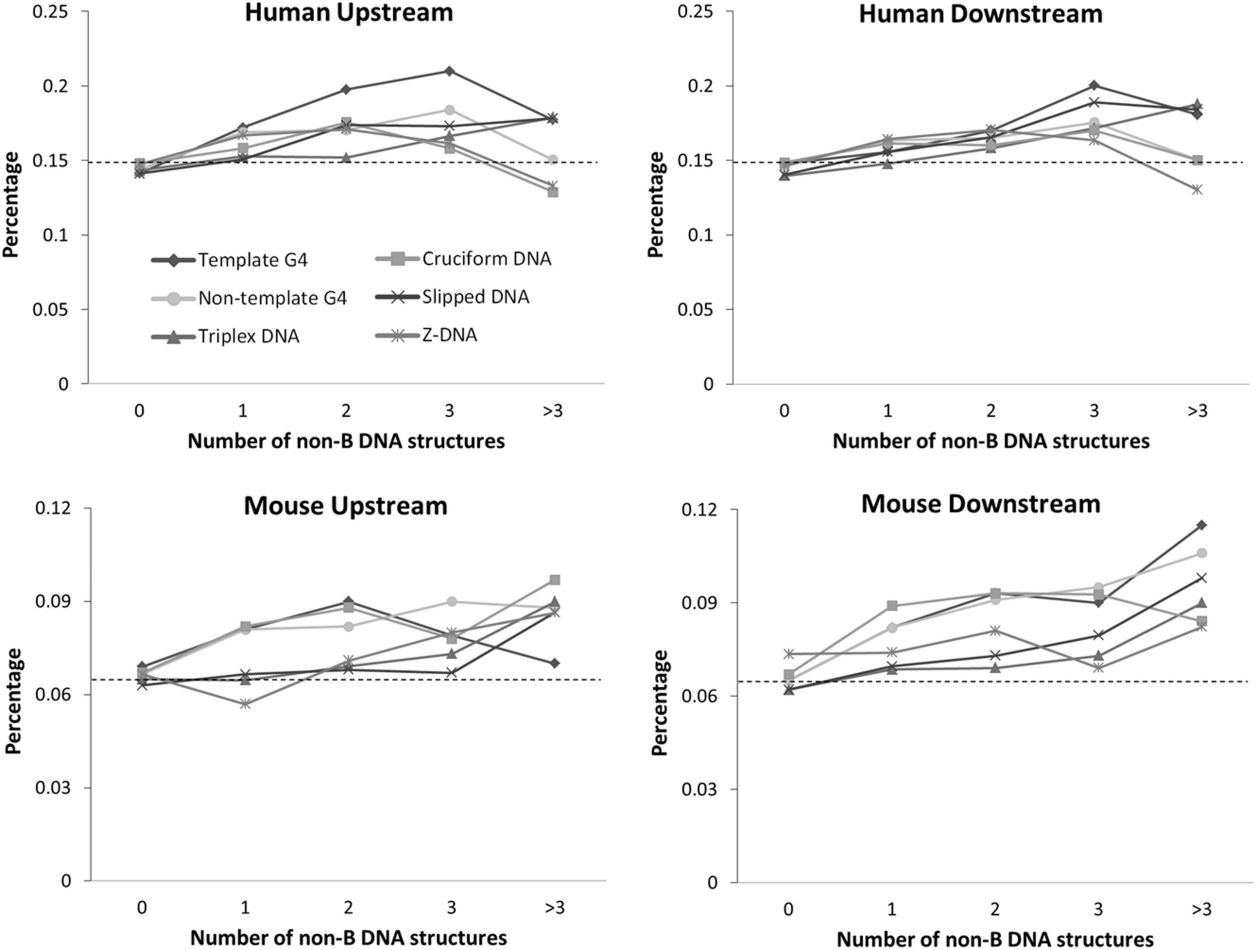
Relationship between non-B DNA structures and exon skipping. The *x*-axis represents the number of each non-B DNA structure in specified genomic loci, and the *y*-axis represents the percentage of observed exon skipping. Background probability is plotted as a horizontal dashed line.

Next, we asked whether the frequency of exon skipping is significantly higher when non-B DNA structures are present. Comparing the proportions of alternative skipped exons with the background exon skipping probability, we found that introns containing non-B DNA occurrences tend to have a higher exon skipping probability than the background, both in human and mouse (Figure 1). Moreover, in agreement with the results from the partial Pearson correlation (Table 2), in human, non-B DNA occurrences located in the upstream tend to have a stronger association with exon skipping. In mouse, however, non-B DNA occurrences located in the downstream have a stronger association (Figure 1). Taken together, presences of non-B DNA structures are associated with exon skipping in a position-specific manner.

Furthermore, we asked whether the proportion of introns having non-B DNA occurrences is significantly higher in alternative than constitutive exons. To avoid potential bias of intron length, here we focused only on the non-B DNA occurrences located within 500 bp upstream or downstream of an exon. Comparing with constitutive exons, we found that for each non-B DNA occurrence in upstream or downstream intron, regardless of in human (Table 3) or mouse (Supplementary Table S1), the proportion is significantly higher in alternative exons than in constitutive exons (*P* < 0.001 by one-sided two-sample proportion test). The results agree with our findings that the presence of non-B DNA occurrences is significantly associated with exon skipping, and hence highlight the importance of non-B DNA structure in the flanking introns of skipped exons.

**Table 3.** Comparison between introns of skipping and constitutive exons that contain non-B DNA structures within 500 bp upstream/downstream region in human

Region	Non-B DNA	Skipping exon (%)^a^	Constitutive exon (%)^b^	*P*-value^c^
Upstream	Template G4	10.42	8.43	1.08 × 10^−22^
	Nontemplate G4	10.19	7.56	5.21 × 10^−42^
	Cruciform DNA	1.17	0.90	5.96 × 10^−5^
	Slipped DNA	6.91	5.82	1.29 × 10^−10^
	Triplex DNA	18.93	18.02	6.55 × 10^−4^
	Z-DNA	4.89	3.79	2.58 × 10^−15^
Downstream	Template G4	8.48	7.83	5.19 × 10^−4^
	Nontemplate G4	6.95	6.36	5.26 × 10^−4^
	Cruciform DNA	1.00	0.77	2.00 × 10^−4^
	Slipped DNA	6.36	5.79	4.72 × 10^−4^
	Triplex DNA	20.00	15.32	3.06 × 10^−70^
	Z-DNA	3.98	3.35	1.04 × 10^−6^

^a^18533 skipping exon in total.

^b^106499 constitutive exon in total.

^c^One-sided two-sample proportion test.

### The relative contributions of non-B DNA occurrences to exon skipping are species-specific

We demonstrated the significant associations between non-B DNA occurrences and exon skipping (Figure 1, Tables 2 and 3), but their relative contributions to exon skipping is still unclear. We therefore applied a multiple logistic regression to assess the effects of the non-B DNA structures and the length of introns to exon skipping (Table 4). The results show that the majority of non-B DNA occurrences (except upstream triplex DNA, downstream G4 and downstream slipped DNA) contribute significantly to exon skipping in human. Particularly, upstream G4 is the most significant. In mouse, however, only downstream G4 and downstream triplex DNA contribute significantly to exon skipping. These results highlight again the position-specific properties of non-B DNA structures in affecting exon skipping, especially G4. In human, G4 is associated with exon skipping only when presented in the upstream intron instead of downstream intron; in mouse, on the other hand, G4 is correlated with exon skipping only when present in the downstream intron. We therefore suggest that the contributions of non-B DNA structures to exon skipping are not only position-specific but also species-specific.

**Table 4.** Relative contributions (regression coefficient of multiple logistic regression) of non-B DNA structures to exon skipping

Position	Predictor variable	Human	Mouse
Upstream	Cruciform DNA	−0.019**	−0.028
	G4	0.029***	0.004
	Slipped DNA	0.009*	0.003
	Triplex DNA	0.001	−2 × 10^−4^
	Z-DNA	−0.018**	−5 × 10^−4^
	Intron length	−2 × 10^−5^	−2 × 10^−4^
Downstream	Cruciform DNA	−0.026**	−0.019
	G4	0.004	0.042***
	Slipped DNA	0.004	4 × 10^−4^
	Triplex DNA	0.007**	0.002*
	Z-DNA	−0.029**	−0.001
	Intron length	6 × 10^−4^	−2 × 10^−5^

Significant regression coefficients (*P* < 0.01) are marked in bold and asterisk.

*P < 0.01, **P < 0.001 and ***P < 0.0001.

### G-run length in G4 as a contributor to exon skipping

**Figure 2.**
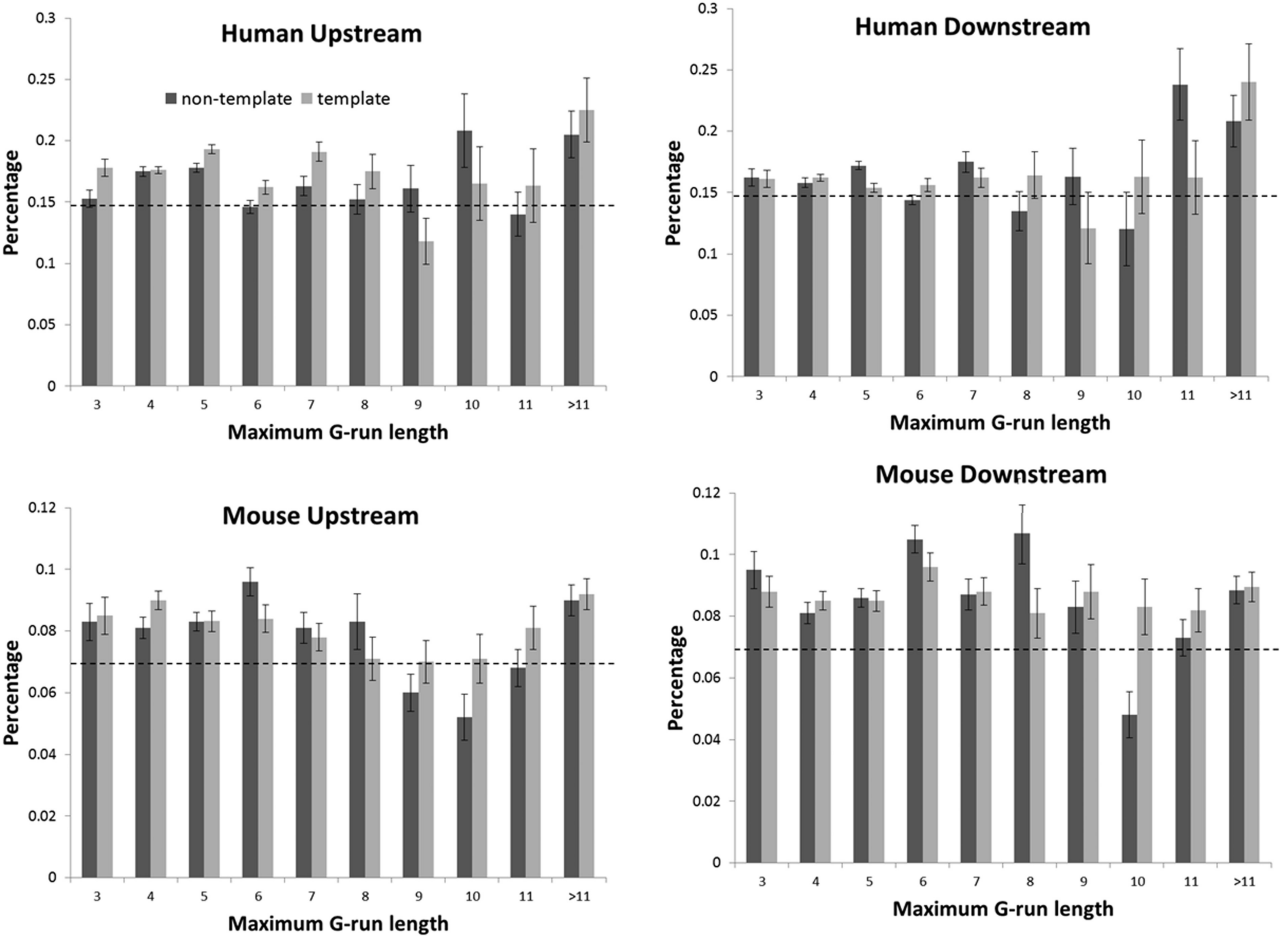
Relationship between the maximum G-run length in G4 and exon skipping. The *x*-axis represents the length of the longest G-run within G4, and the *y*-axis represents the percentage of observed exon skipping. Background probability is plotted as a horizontal dashed line.

To examine the relationship between G4 and exon skipping in detail, we further analyzed G-run. G-run length has been shown experimentally to be important in enhancing formation rate and stabilizing structure of G4 (30). Shortening of G-run length dramatically reduces the melting temperature of G4 (30). Therefore, it is interesting to examine the relationship between G-run length and exon skipping activity. We identified G4-containing cassette exons and grouped them based on the G-run occurrence strand, region and maximum G-run length. We found that most of the G4-containing cassette exons have higher exon skipping proportion than the background exon skipping probability in both human and mouse (Figure 2). Moreover, significant correlation between G-run length and exon skipping was observed (Figure 2, partial Pearson correlation test, *P* = 3.5 × 10^−^^11^). Interestingly, when the G-run length is nine on the template strand, a drop below the background level was observed in human (in mouse the drop occurs when G-run length is 10 on the nontemplate strand).

## DISCUSSION

We examined potential roles of five non-B DNA structures (G4, cruciform DNA, triplex DNA, slipped DNA and Z-DNA) that have the capability to impede transcription (26–28) in splicing regulation. Our results indicate that, in addition to the well-known RNA and protein structure, non-B DNA structures may also contribute significantly to exon skipping, thus providing a new perspective for studying the regulation of alternative splicing. We also built-on and broadened the understandings of the relationship between alternative splicing and G4 (19,20). Moreover, we found that specific region of non-B DNA structures (Figure 1) and length of G-run length (Figure 2) are particularly important in determining the contributions of non-B DNA structures to exon skipping.

It has been suggested that G4 could block Pol II activity during transcription elongation (15). Also, G-runs have long been identified to be an important sequence element in both DNA and RNA, which interact with splicing factors, such as heterogeneous nuclear ribonucleoprotein (hnRNP) H and hnRNP F family (36–40). Moreover, the occurrence of a G4 motif may correspond to the formation of G4 structures not only in DNA, but also possibly in RNA (41). It is therefore not difficult to conjecture that G4s may serve dual roles, as structural blockages for Pol II and recognition sites for mRNA splicing factors. Interestingly, recent studies indicated that R-loop (RNA–DNA hybrid structure characterized by G-rich nascent RNA base pairing with a C-rich template strand of DNA) plays a similar role in exacerbating transcription blockage (42) and is involved in alternative splicing (43). Despite these advances, the mystery of their functions *in vivo* remains unresolved. A challenge here is that R-loop and G4 share a similar sequence property, i.e. G-rich, therefore it is computationally difficult to distinguish one from the other solely from sequence-based analysis or even with experimental approaches. Another caveat in this study involves the use of sequence-based motifs in our computational prediction of structural occurrences. Current understanding of G4 blockage revolves around its 3D structure. However, the use of 3D structure is difficult in a genome-wide *in silico* study because kinetics of structural formation differs between cell types and developmental stages. Predicted Z-DNA loci and the binding loci of Zα domain of ADAR1, which displays high specificity for Z-DNA, only partially overlap (44). Precise prediction requires immense amount of data, including chromatin modification, supercoiling, replication, repair and transcription, which, collectively, is currently unavailable. We thus compromised by using sequence motifs because non-B DNA structures have well-characterized motifs, and some of them are directly defined by their sequence motifs (45). Therefore, while the 3D structure occurrence was not experimentally identified, motif-based prediction is still a good reflection of the presence of the actual structures.

In addition to G4, triplex DNA is another non-B DNA structure that consistently shows significant associations to exon skipping in most of the positions in human (Table 2). In fact, a study has indicated that triplex is involved in RNA processing (46). Although the effect of triplex DNA on alternative splicing is still unclear, five alternative splicing-related hnRNP proteins (47) have been found experimentally to interact with triplex DNA (48). Moreover, a recent study suggests that intron self-splicing may be associated with enzymatic triplex DNA (49). The formation of triplex DNA could even prevent binding and activation of RNA-dependent protein kinase, and subsequently influence the synthesis rate of proteins (50,51). In addition to DNA structures, which could inhibit Pol II, recent evidence also suggests a role for higher-order chromatin structure in splicing outcomes (5). Alló *et al.* hypothesized that particular chromatin structures may prevent Pol II elongation, and the delay in elongation could, in turn, provide more time for the recruitment of splicing factors and recognition of a splice site. Their results demonstrate that higher levels of heterochromatin marks, which are triggered by small interfering RNAs, could result in alternative splicing (5). Those observations support the kinetic model hypothesis of the association between alternative splicing and the dynamics of elongation rate.

Taken together, our work introduced the general non-B DNA structure in alternative splicing, and laid grounds for investigation on the associations of non-B DNA structures with exon skipping. Moreover, their contribution to exon skipping may be heavily influenced by occurrence region, which are spatially important for any structural interactions. We suggested that as Pol II is transcribing, non-B DNA structures represent structural obstacles that hamper the transcription process, and eventually result in exon skipping. In fact, non-B DNA structures that occurred closer to exons tend to lead a higher possibility to exon skipping (Details in Supplementary Figure S1). As the proportion of introns having non-B DNA occurrences is higher in alternative than constitutive exons (Table 3), the majority of the proportion differences also are increasing when approaching exons (Details in Supplementary Figure S2). These observations further support the kinetic model hypothesis, and imply that non-B DNA structures, which affect exon skipping, may tend to occur close to the exons. Moreover, there are positions in which non-B DNA structures have significant potential to interfere with exon skipping, while some positions lack the potential to affect exon skipping. From the perspectives of the kinetic model, the insignificance under particular positions may be due to (i) the structures lacking the ability to impede Pol II during elongation, or (ii) those non-B DNA structures being transiently formed *in vivo*. Further experimental studies may be required to examine the suggested association of non-B DNA structures in exon skipping, as well as to elucidate underlying mechanisms.

Another possible explanation to the identified association is that specific proteins may recognize non-B DNA motifs, instead of non-B DNA structure, and then influence or interact with splicing factors. For example, the occurrences of Z-DNA motif in the human and mouse genomes are usually AC/TG repeats, which could be recognized by DNA-binding proteins, such as RAP1 and NDT80, in which the binding motifs are AC/TG-rich. However, our preliminary analysis of six AC/TG-rich motifs merely shows weak enrichments within identified Z-DNA regions (Details in Supplementary Table S2). To the best of our knowledge, there is no current literature or experiments that provide evidence on proteins that influence alternative splicing through binding to non-B DNA motifs. We nonetheless believe further experiments, such as chromatin immunoprecipitation experiments, may provide advance information to reveal the proposed scenario.

While our hypothesis specifically focuses on the kinetic coupling model of co-transcriptional splicing, non-B DNA structures may also have an impact under the recruitment coupling model, which has been extensively studied and suggested to regulate alternative splicing (1,52–55). The presence of non-B DNA structure may alter the chromosomal state, and thus lead to a spatial hindrance that affects the recruitment of splicing factors to the RNA polymerase complex. In fact, the formation of non-B DNA structure is influenced by local superhelical density, transcriptional status and chromatin factors, which are temporal and condition-specific (56). For example, negative supercoiling has been demonstrated to assist the structural transition from B-DNA to non-B DNA (57,58), which vary depending on chromosomal state and transcriptional process (59). Accordingly, it is reasonable that non-B DNA structures form dynamically according to the changing environments. The current model we proposed is under the assumption that non-B DNA motifs would form non-B DNA structures under unchanging condition, ignoring the potential dynamic and temporal effects from regulatory and chromosomal factors. Although this assumption may possibly reduce the significance of our model, our investigation was limited by the incomplete and the scarcely available genome-wide data of the loci of non-B DNA structure *in vivo* and in dynamic conditions. Nevertheless, we anticipate that these computational results will bring insights to the functionalities of non-B DNA structures. Future models that illustrate a more comprehensive picture of how non-B DNA structures impact alternative splicing can be addressed as more integrative data on the global and dynamic information of chromosomal factors and regulators become available.

## SUPPLEMENTARY DATA

Supplementary Data are available at NAR Online, including [60–62].

## FUNDING

National Science Council of Taiwan [NSC100-2628-E-001-006-MY3 to H.-K.T.]. Funding for open access charge: National Science Council of Taiwan.

*Conflict of interest statement*. None declared.

## Supplementary Material

Supplementary Data

## References

[1] Kornblihtt AR, Schor IE, Alló M, Dujardin G, Petrillo E, Muñoz MJ (2013). Alternative splicing: a pivotal step between eukaryotic transcription and translation. Nat. Rev. Mol. Cell Biol..

[2] Muñoz MJ, de la Mata M, Kornblihtt AR (2010). The carboxy terminal domain of RNA polymerase II and alternative splicing. Trends Biochem. Sci..

[3] De la Mata M, Kornblihtt AR (2006). RNA polymerase II C-terminal domain mediates regulation of alternative splicing by SRp20. Nat. Struct. Mol. Biol..

[4] Monsalve M, Wu Z, Adelmant G, Puigserver P, Fan M, Spiegelman BM (2000). Direct coupling of transcription and mRNA processing through the thermogenic coactivator PGC-1. Mol. Cell.

[5] Alló M, Buggiano V, Fededa JP, Petrillo E, Schor I, de la Mata M, Agirre E, Plass M, Eyras E, Elela SA (2009). Control of alternative splicing through siRNA-mediated transcriptional gene silencing. Nat. Struct. Mol. Biol..

[6] de la Mata M, Alonso CR, Fededa JP, Pelisch F, Cramer P, Bentley D, Kornblihtt AR (2003). A slow RNA polymerase II affects alternative splicing *in vivo*. Mol. Cell.

[7] Howe KJ, Kane CM, Ares M (2003). Perturbation of transcription elongation influences the fidelity of internal exon inclusion in *Saccharomyces cerevisiae*. RNA.

[8] Roberts GC, Gooding C, Mak HY, Proudfoot NJ, Smith CW (1998). Co-transcriptional commitment to alternative splice site selection. Nucleic Acids Res..

[9] Nogués G, Muñoz MJ, Kornblihtt AR (2003). Influence of polymerase II processivity on alternative splicing depends on splice site strength. J. Biol. Chem..

[10] Ip JY, Schmidt D, Pan Q, Ramani AK, Fraser AG, Odom DT, Blencowe BJ (2011). Global impact of RNA polymerase II elongation inhibition on alternative splicing regulation. Genome Res..

[11] Hershman SG, Chen Q, Lee JY, Kozak ML, Yue P, Wang L-S, Johnson FB (2008). Genomic distribution and functional analyses of potential G-quadruplex-forming sequences in *Saccharomyces cerevisiae*. Nucleic Acids Res..

[12] Siddiqui-Jain A, Grand CL, Bearss DJ, Hurley LH (2002). Direct evidence for a G-quadruplex in a promoter region and its targeting with a small molecule to repress c-MYC transcription. Proc. Natl Acad. Sci. USA.

[13] Bacolla A, Wojciechowska M, Kosmider B, Larson JE, Wells RD (2006). The involvement of non-B DNA structures in gross chromosomal rearrangements. DNA Repair.

[14] Bochman ML, Paeschke K, Zakian VA (2012). DNA secondary structures: stability and function of G-quadruplex structures. Nat. Rev. Genet..

[15] Tornaletti S, Park-Snyder S, Hanawalt PC (2008). G4-forming sequences in the non-transcribed DNA strand pose blocks to T7 RNA polymerase and mammalian RNA polymerase II. J. Biol. Chem..

[16] Huppert JL, Bugaut A, Kumari S, Balasubramanian S (2008). G-quadruplexes: the beginning and end of UTRs. Nucleic Acids Res..

[17] Alexander RD, Innocente Sa, Barrass JD, Beggs JD (2010). Splicing-dependent RNA polymerase pausing in yeast. Mol. Cell.

[18] Carrillo Oesterreich F, Preibisch S, Neugebauer KM (2010). Global analysis of nascent RNA reveals transcriptional pausing in terminal exons. Mol. Cell.

[19] Kostadinov R, Malhotra N, Viotti M, Shine R, D’Antonio L, Bagga P (2006). GRSDB: a database of quadruplex forming G-rich sequences in alternatively processed mammalian pre-mRNA sequences. Nucleic Acids Res..

[20] Marcel V, Tran PLT, Sagne C, Martel-Planche G, Vaslin L, Teulade-Fichou M-P, Hall J, Mergny J-L, Hainaut P, Van Dyck E (2011). G-quadruplex structures in TP53 intron 3: role in alternative splicing and in production of p53 mRNA isoforms. Carcinogenesis.

[21] He Y, Neumann RD, Panyutin IG (2004). Intramolecular quadruplex conformation of human telomeric DNA assessed with 125I-radioprobing. Nucleic Acids Res..

[22] Webber AL, Masiero S, Pieraccini S, Burley JC, Tatton AS, Iuga D, Pham TN, Spada GP, Brown SP (2011). Identifying guanosine self assembly at natural isotopic abundance by high-resolution 1H and 13C solid-state NMR spectroscopy. J. Am. Chem. Soc..

[23] Kuryavyi V, Patel DJ (2010). Solution structure of a unique G-quadruplex scaffold adopted by a guanosine-rich human intronic sequence. Structure.

[24] Ho PS (1994). The non-B-DNA structure of d(CA/TG)n does not differ from that of Z-DNA. Proc. Nat. Acad. Sci. USA.

[25] Peck LJ, Wang JC (1983). Energetics of B-to-Z transition in DNA. Proc. Natl Acad. Sci. USA.

[26] Cerná A, Cuadrado A, Jouve N, Díaz de la Espina SM, De la Torre C (2004). Z-DNA, a new in situ marker for transcription. Eur. J. Histochem..

[27] Brázda V, Laister RC, Jagelská EB, Arrowsmith C (2011). Cruciform structures are a common DNA feature important for regulating biological processes. BMC Mol. Biol..

[28] Belotserkovskii BP, De Silva E, Tornaletti S, Wang G, Vasquez KM, Hanawalt PC (2007). A triplex-forming sequence from the human c-MYC promoter interferes with DNA transcription. J. Biol. Chem..

[29] Salinas-Rios V, Belotserkovskii BP, Hanawalt PC (2011). DNA slip-outs cause RNA polymerase II arrest *in vitro*: potential implications for genetic instability. Nucleic Acids Res..

[30] Gros J, Rosu F, Amrane S, De Cian A, Gabelica V, Lacroix L, Mergny J-L (2007). Guanines are a quartet’s best friend: impact of base substitutions on the kinetics and stability of tetramolecular quadruplexes. Nucleic Acids Res..

[31] Koscielny G, Le Texier V, Gopalakrishnan C, Kumanduri V, Riethoven J-J, Nardone F, Stanley E, Fallsehr C, Hofmann O, Kull M (2009). ASTD: The Alternative Splicing and Transcript Diversity database. Genomics.

[32] Cer RZ, Donohue DE, Mudunuri US, Temiz Na, Loss Ma, Starner NJ, Halusa GN, Volfovsky N, Yi M, Luke BT (2013). Non-B DB v2.0: a database of predicted non-B DNA-forming motifs and its associated tools. Nucleic Acids Res..

[33] Lodish H, Berk A, Matsudaira P, Kaiser C, Krieger M, Scott M, Zipursky S, Darnell J (2004). Molecular Cell Biology.

[34] Bell MV, Cowper AE, Lefranc M, Bell JI, Screaton GR (1998). Influence of intron length on alternative splicing of CD44 influence of intron length on alternative splicing of CD44. Mol.Cell. Biol..

[35] Fox-Walsh KL, Dou Y, Lam BJ, Hung S-P, Baldi PF, Hertel KJ (2005). The architecture of pre-mRNAs affects mechanisms of splice-site pairing. Proc. Natl Acad. Sci. USA.

[36] Dominguez C, Allain FH-T (2006). NMR structure of the three quasi RNA recognition motifs (qRRMs) of human hnRNP F and interaction studies with Bcl-x G-tract RNA: a novel mode of RNA recognition. Nucleic Acids Res..

[37] Expert-Bezançon A, Sureau A, Durosay P, Salesse R, Groeneveld H, Lecaer JP, Marie J (2004). hnRNP A1 and the SR proteins ASF/SF2 and SC35 have antagonistic functions in splicing of beta-tropomyosin exon 6B. J. Biol. Chem..

[38] Grabowski PJ (2004). A molecular code for splicing silencing: configurations of guanosine-rich motifs. Biochem. Soc. Transact..

[39] Hai Y, Cao W, Liu G, Hong S-P, Elela SA, Klinck R, Chu J, Xie J (2008). A G-tract element in apoptotic agents-induced alternative splicing. Nucleic Acids Res..

[40] Marcucci R, Baralle FE, Romano M (2007). Complex splicing control of the human Thrombopoietin gene by intronic G runs. Nucleic Acids Res..

[41] Du X, Wojtowicz D, Bowers AA, Levens D, Benham CJ, Przytycka TM (2013). The genome-wide distribution of non-B DNA motifs is shaped by operon structure and suggests the transcriptional importance of non-B DNA structures in *Escherichia coli*. Nucleic Acids Res..

[42] Belotserkovskii BP, Liu R, Tornaletti S, Krasilnikova MM (2010). Mechanisms and implications of transcription blockage by guanine-rich DNA sequences. Proc. Natl Acad. Sci. USA.

[43] Wongsurawat T, Jenjaroenpun P, Kwoh CK, Kuznetsov V (2012). Quantitative model of R-loop forming structures reveals a novel level of RNA-DNA interactome complexity. Nucleic Acids Res..

[44] Li H, Xiao J, Li J, Lu L, Feng S, Dröge P (2009). Human genomic Z-DNA segments probed by the Z alpha domain of ADAR1. Nucleic Acids Res..

[45] Bacolla A, Wells RD (2004). Non-B DNA conformations, genomic rearrangements, and human disease. J. Biol. Chem..

[46] Buske Fa, Mattick JS, Bailey TL (2011). Potential *in vivo* roles of nucleic acid triple-helices. RNA Biol..

[47] Chen M, Zhang J, Manley JL (2010). Turning on a fuel switch of cancer: hnRNP proteins regulate alternative splicing of pyruvate kinase mRNA. Cancer Res..

[48] Guillonneau F, Guieysse aL, Le Caer JP, Rossier J, Praseuth D (2001). Selection and identification of proteins bound to DNA triple-helical structures by combination of 2D-electrophoresis and MALDI-TOF mass spectrometry. Nucleic Acids Res..

[49] Toor N, Keating KS, Pyle AM (2009). Structural insights into RNA splicing. Curr. Opin. Struct. Biol..

[50] Vuyisich M, Beal PA (2000). Regulation of the RNA-dependent protein kinase by triple helix formation. Nucleic Acids Res..

[51] Jaramillo ML, Abraham N, Bell JC (1995). The interferon system: a review with emphasis on the role of PKR in growth control. Cancer Invest..

[52] Martins SB, Rino J, Carvalho T, Carvalho C, Yoshida M, Klose JM, de Almeida SF, Carmo-Fonseca M (2011). Spliceosome assembly is coupled to RNA polymerase II dynamics at the 3’ end of human genes. Nat. Struct. Mol. Biol..

[53] Lee K, Tarn WY (2013). Coupling pre-mRNA processing to transcription on the RNA factory assembly line. RNA Biol..

[54] Kosti I, Radivojac P, Mandel-Gutfreund Y (2012). An integrated regulatory network reveals pervasive cross-regulation among transcription and splicing factors. PLoS Comput. Biol..

[55] Shukla S, Oberdoerffer S (2012). Co-transcriptional regulation of alternative pre-mRNA splicing. Biochim. Biophys. Acta.

[56] Wells RD (2007). Non-B DNA conformations, mutagenesis and disease. Trends Biochem. Sci..

[57] Kouzine F, Sanford S, Elisha-Feil Z, Levens D (2008). The functional response of upstream DNA to dynamic supercoiling *in vivo*. Nat. Struct. Mol. Biol..

[58] Brooks Ta, Hurley LH (2009). The role of supercoiling in transcriptional control of MYC and its importance in molecular therapeutics. Nat. Rev. Cancer.

[59] Kramer PR, Sinden RR (1997). Measurement of unrestrained negative supercoiling and topological domain size in living human cells. Biochemistry.

[60] Mahony S, Benos PV (2007). STAMP: a web tool for exploring DNA-binding motif similarities. Nucleic Acids Res..

[61] Bryne JC, Valen E, Tang M-HE, Marstrand T, Winther O, da Piedade I, Krogh A, Lenhard B, Sandelin A (2008). JASPAR, the open access database of transcription factor-binding profiles: new content and tools in the 2008 update. Nucleic Acids Res..

[62] Turatsinze J-V, Thomas-Chollier M, Defrance M, van Helden J (2008). Using RSAT to scan genome sequences for transcription factor binding sites and cis-regulatory modules. Nat. Protoc..

