# Supplementary material for: Associations between intronic non-B DNA structures and exon skipping
Source: Nucleic Acids Res. 2013 Oct 22;42(2):739–47. doi: 10.1093/nar/gkt939 (PMC3902930; doi:10.1093/nar/gkt939)
Supplement: Supplementary Data [file supp_42_2_739__index.html]

Associations between intronic non-B DNA structures and exon skipping — Associations between intronic non-B DNA structures and exon skipping — Supplementary Data 

# Associations between intronic non-B DNA structures and exon skipping

## Supplementary Data

files

**Files in this Data Supplement:**

- Supplementary Data - docx file
