# Supplementary material for: Associations between intronic non-B DNA structures and exon skipping
Source: Nucleic Acids Res. 2013 Oct 22;42(2):739–47. doi: 10.1093/nar/gkt939 (PMC3902930; doi:10.1093/nar/gkt939)
Supplement: Supplementary Data [file supp_gkt939_nar-01986-f-2013-File004.docx]

**SUPPLEMENTARY DATA**

**Table S1. Comparison between introns of skipping and constitutive exons that contains non-B DNA structures within 500 bp upstream/downstream region in mouse**

| Region | Non-B DNA | Skipping exon (%)^1^ | Constitutive exon (%)^2^ | *P*-value^3^ |
| --- | --- | --- | --- | --- |
| Upstream | Template G4 | 5.86 | 4.81 | 1.00×10^-6^ |
|  | Non-template G4 | 5.76 | 5.01 | 4.45×10^-4^ |
|  | Cruciform DNA | 1.74 | 1.26 | 1.71×10^-5^ |
|  | Slipped DNA | 17.33 | 16.13 | 7.83×10^-4^ |
|  | Triplex DNA | 21.07 | 18.84 | 1.52×10^-8^ |
|  | Z-DNA | 10.52 | 9.48 | 2.92×10^-4^ |
| Downstream | Template G4 | 5.83 | 4.94 | 3.53×10^-5^ |
|  | Non-template G4 | 5.96 | 5.04 | 2.35×10^-5^ |
|  | Cruciform DNA | 1.94 | 1.26 | 1.94×10^-9^ |
|  | Slipped DNA | 17.17 | 15.96 | 6.82×10^-4^ |
|  | Triplex DNA | 21.53 | 18.84 | 1.16×10^-11^ |
|  | Z-DNA | 10.86 | 9.89 | 8.28×10^-4^ |

^1^ 9,477 skipping exon in total

^2^ 127,372 constitutive exon in total

^3^ One-sided two-sample proportion test

**Table S2. Enrichments of AC/TG-rich motifs around Z-DNA**

Six human transcription factors in which the binding motifs are identified as AC/TG-rich motifs by STAMP (1) and the JASPAR database (2). We scanned these motifs within [-50, +50] region of Z-DNA (by the tool matrix-scan in RSAT (3) with default settings), and calculated the significances of motif enrichment within the Z-DNA region, comparing with random sequence (one-sided two-sample proportion test).

| Protein name | Binding motif | *P*-value^1^ |
| --- | --- | --- |
| RAP1 | 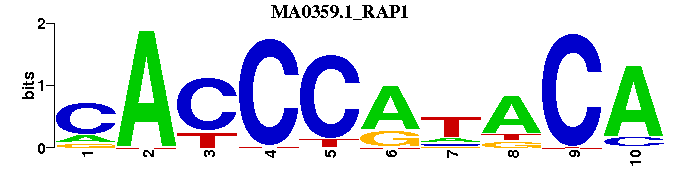 | 3.1×10^-2^ |
| NDT80 | 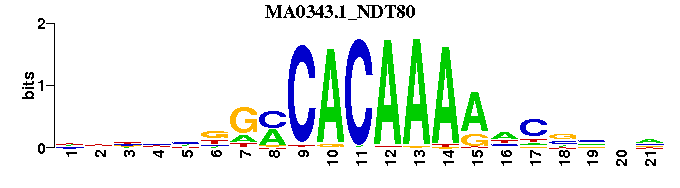 | 1.8×10^-2^ |
| EGR1 | 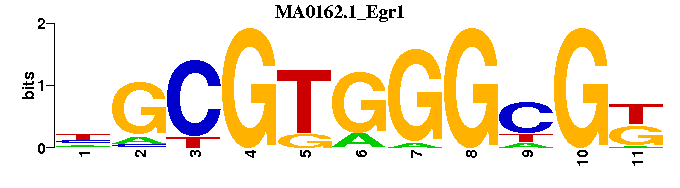 | 8.4×10^-2^ |
| FOXI1 | 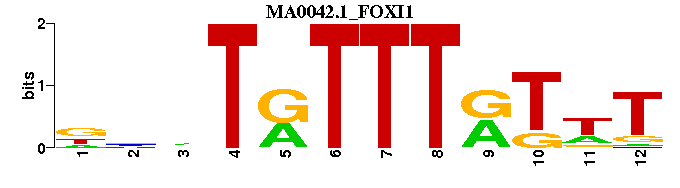 | 0.10 |
| FOXD3 | 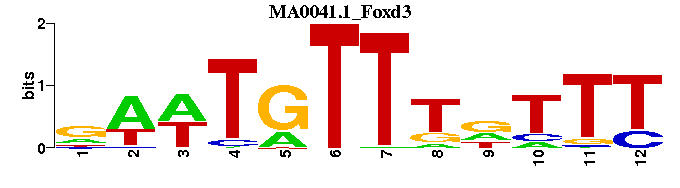 | 0.11 |
| MATA1 | 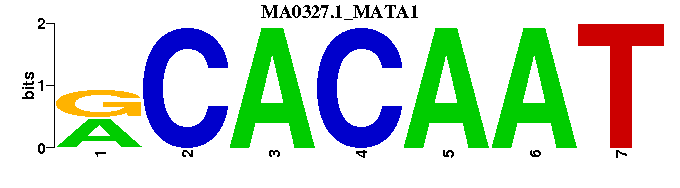 | 0.67 |

^1^ One-sided two-sample proportion test

| **Upstream intron** | **Downstream intron** |
| --- | --- |
| 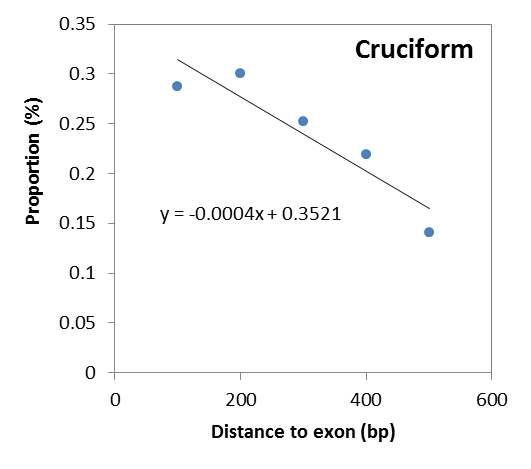 | 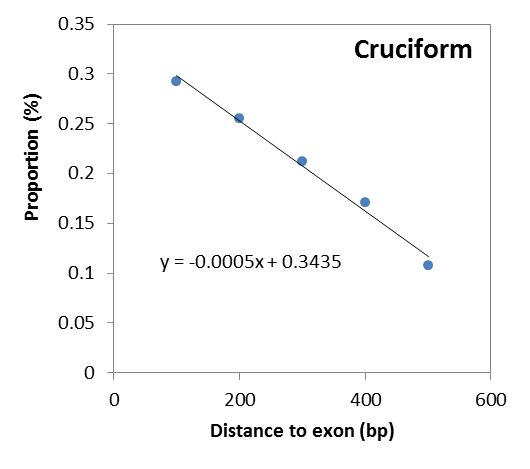 |
| 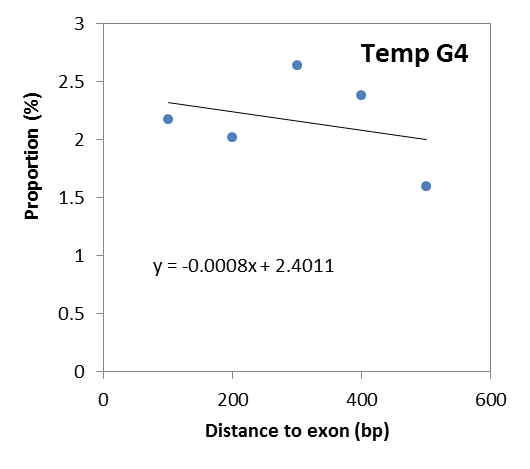 | 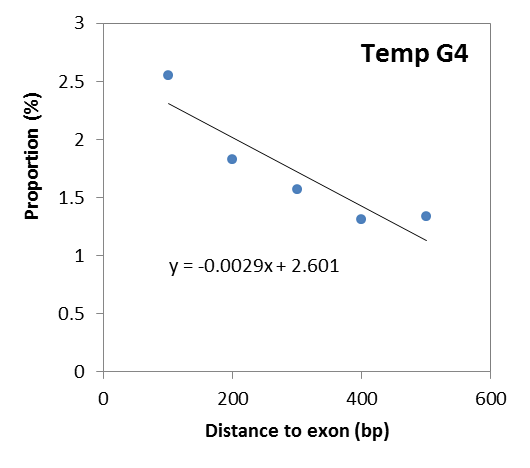 |
| 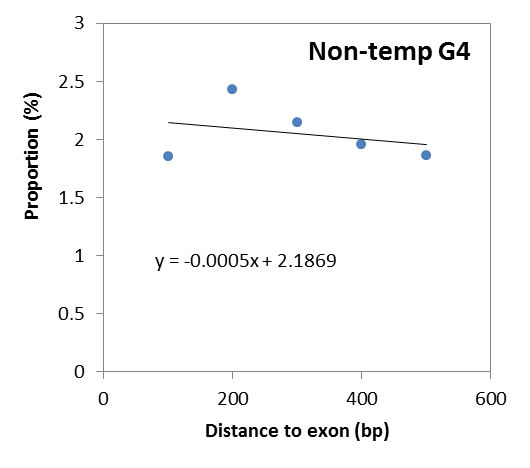 | 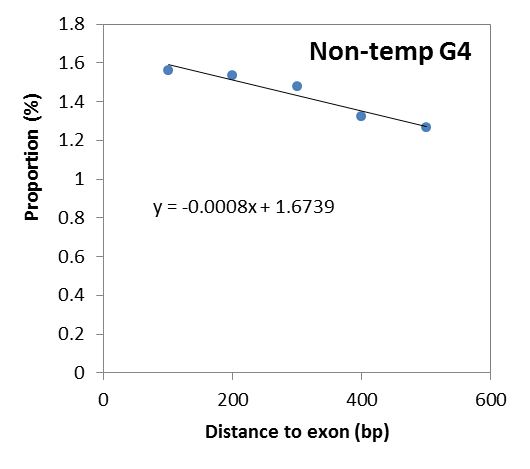 |

**Figure S1. The proportion of intron containing non-B structure occurrences in human skipped exons.** To control effect of intron length, only 500 bp upstream/downstream regions were examined. We then calculated the proportion of containing non-B structure occurrences for each 100 bp bin. Each blue dot indicates the proportion. The black line indicates a linear regression line, and the regression function is also shown.

| **Upstream intron** | **Downstream intron** | |
| --- | --- | --- |
| 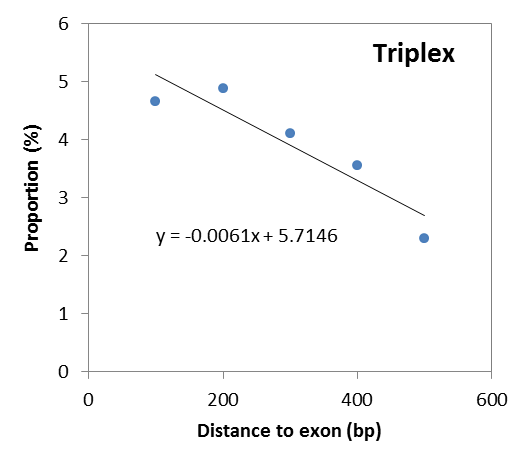 | | 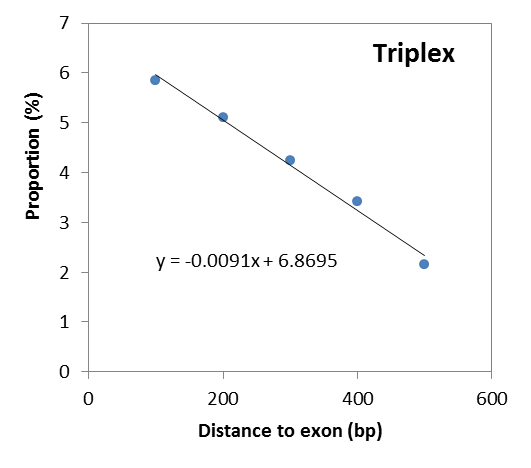 |
| 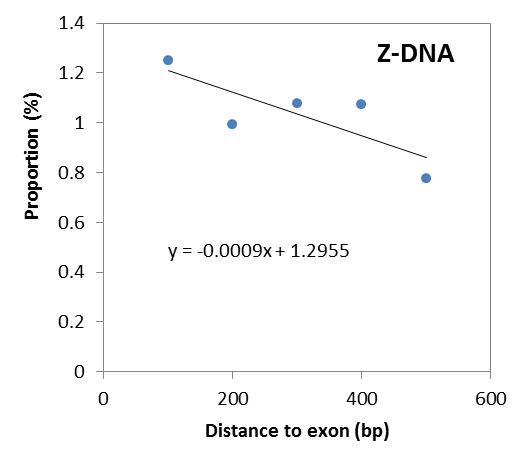 | | 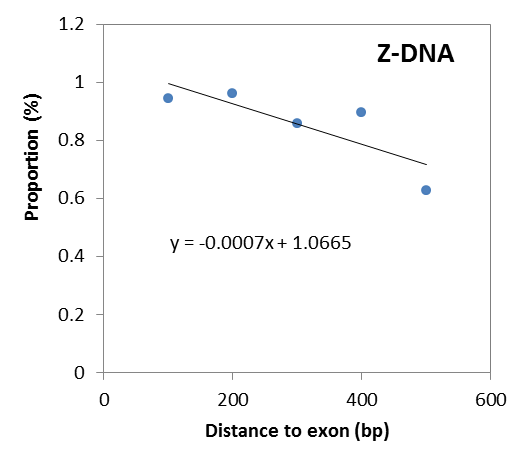 |
| 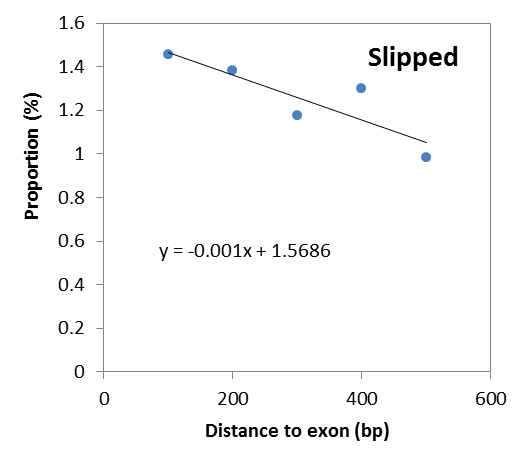 | | 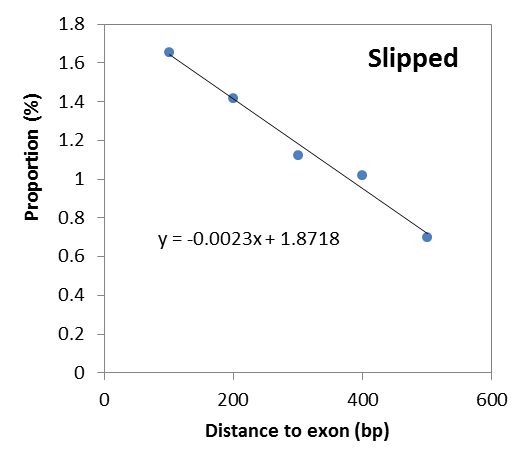 |

**Figure S1 (continued).** (Caption shown on previous page)

| **Upstream intron** | **Downstream intron** |
| --- | --- |
| 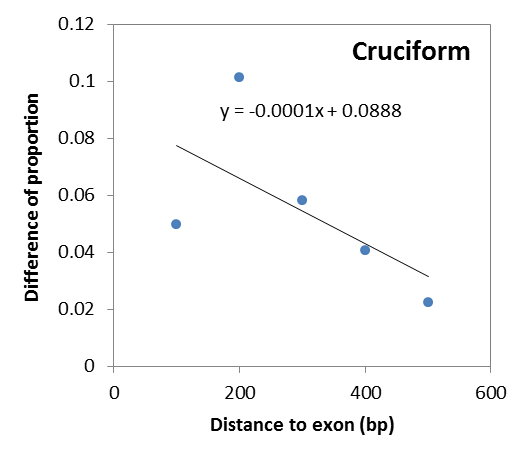 | 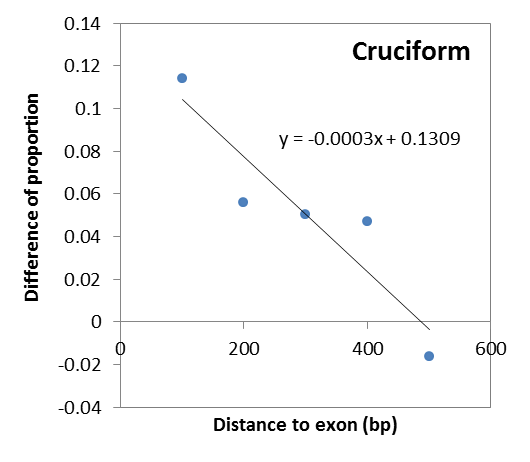 |
| 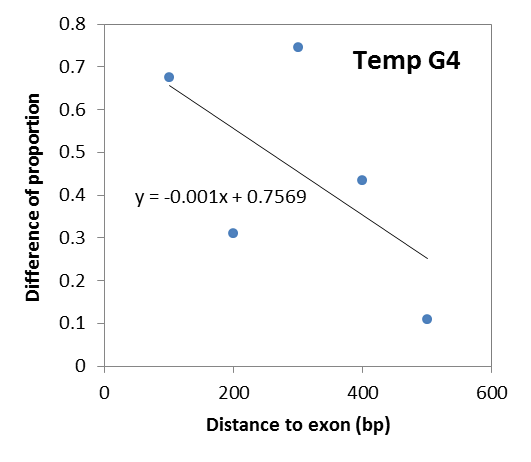 | 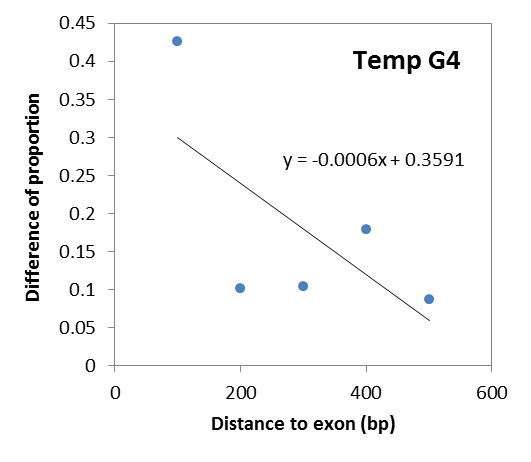 |
| 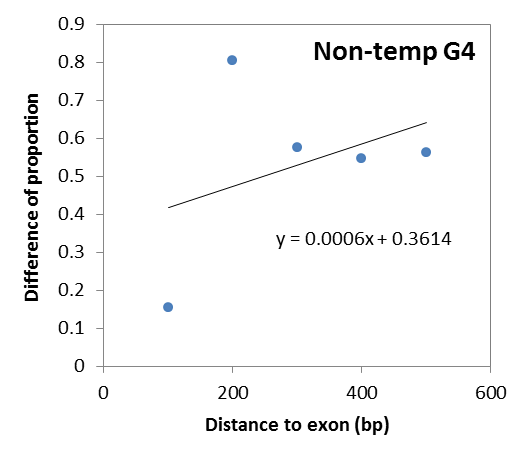 | 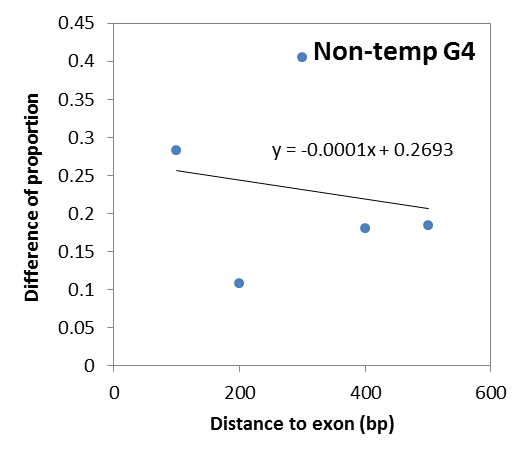 |

**Figure S2. The difference between alternative and constitutive exons in the proportion of intron containing non-B structure occurrences in human.** For both alternative and constitutive exons, we calculated the proportion of introns containing non-B structure occurrences for each 100 bp bin. Each blue dot indicates the difference between the proportions for alternative and constitutive exons. The black line indicates a linear regression line, and the regression function is also shown.

| **Upstream intron** | **Downstream intron** | |
| --- | --- | --- |
| 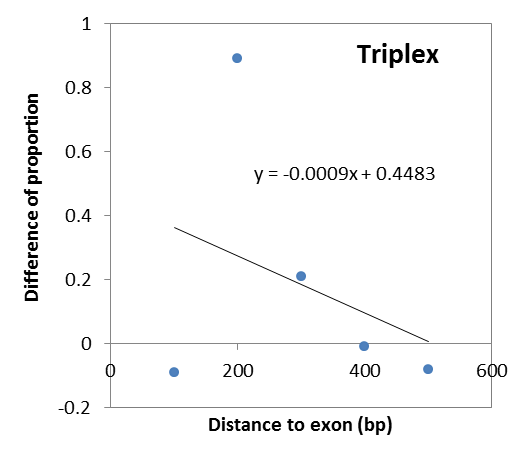 | | 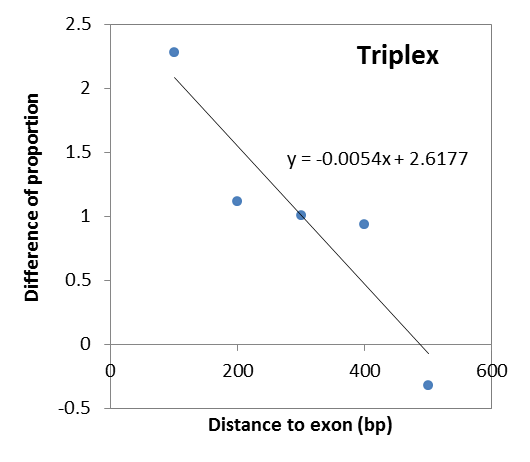 |
| 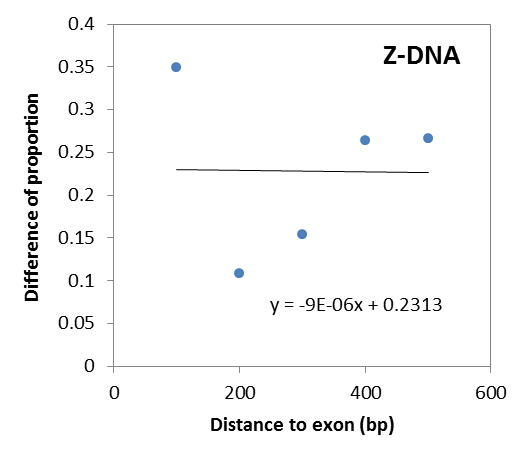 | | 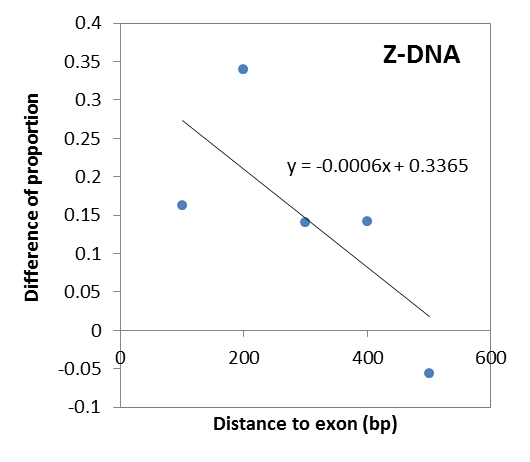 |
| 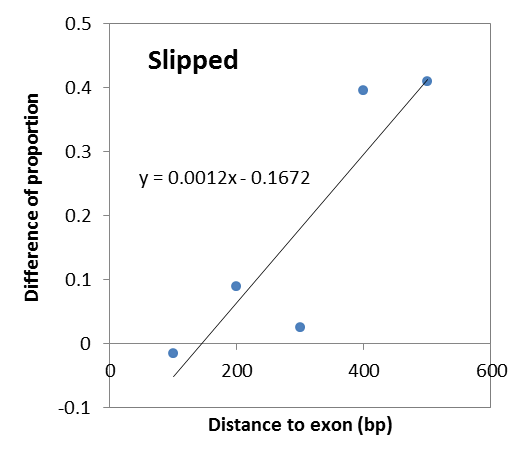 | | 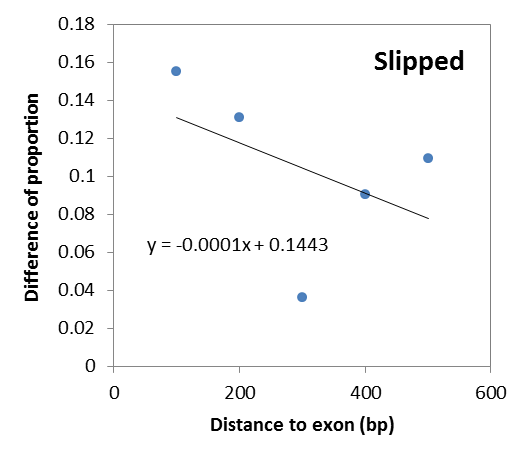 |

**Figure S2 (continued).** (Caption shown on previous page)

**Reference**

1. Mahony,S. and Benos,P. V (2007) STAMP: a web tool for exploring DNA-binding motif similarities. *Nucleic acids research*, **35**, W253–8.

2. Bryne,J.C., Valen,E., Tang,M.-H.E., Marstrand,T., Winther,O., da Piedade,I., Krogh,A., Lenhard,B. and Sandelin,A. (2008) JASPAR, the open access database of transcription factor-binding profiles: new content and tools in the 2008 update. *Nucleic acids research*, **36**, D102–6.

3. Turatsinze,J.-V., Thomas-Chollier,M., Defrance,M. and van Helden,J. (2008) Using RSAT to scan genome sequences for transcription factor binding sites and cis-regulatory modules. *Nature protocols*, **3**, 1578–88.
